# Fatigue in children and adolescents with inflammatory bowel disease: a cross-sectional study

**DOI:** 10.3389/fped.2024.1519779

**Published:** 2024-12-19

**Authors:** Yahui Zuo, Jinjin Cao, Yuanyuan Wang, Wenqian Cai, Mei Li

**Affiliations:** ^1^ Department of Nursing, Children’s Hospital of Nanjing Medical University, Nanjing, China; ^2^ School of Nursing, Nanjing Medical University, Nanjing, China; ^3^ Department of Nursing, Nanjing BenQ Medical Center, The Affiliated BenQ Hospital of Nanjing Medical University, Nanjing, China; ^4^ School of Pediatrics, Nanjing Medical University, Nanjing, China; ^5^ Department of Intensive Care Unit, The First Affiliated Hospital of Zhejiang Chinese Medical University (Zhejiang Provincial Hospital of Chinese Medicine), Hangzhou, China

**Keywords:** adolescents, children, fatigue, inflammatory bowel disease, influencing factors

## Abstract

**Background:**

In recent years, there has been an observed increase in the global diagnosis rate of inflammatory bowel disease among children and adolescents. In inactive disease states, fatigue has emerged as the most debilitating symptom, while in active disease states, it ranks second. However, there remains a notable lack of understanding regarding fatigue in pediatric inflammatory bowel disease patients. Consequently, this study sought to investigate the prevalence and status of fatigue in a cohort of pediatric inflammatory bowel disease patients in China.

**Methods:**

A cross-sectional, questionnaire-based survey was conducted. The researchers recruited 110 patients with inflammatory bowel disease using the convenient sampling method between 1 September 2021 until 31 November 2022 in Department of Gastroenterology of Children's Hospital of Nanjing Medical University. Fatigue was assessed using the Multidimensional Fatigue Scale, while potential factors associated with fatigue were analyzed through univariate and multiple regression analyses.

**Results:**

The study found that the total score of fatigue in children with inflammatory bowel disease was 62.22 ± 20.55. The univariate analysis revealed significant differences in the degree of fatigue across regions, ages, disease severities, corticosteroid use, and biological agent use. Furthermore, the multiple regression analysis indicated a significant difference in BMI (*p* < 0.05).

**Conclusions:**

Fatigue is a multidimensional symptom that affects a majority of pediatric inflammatory bowel disease patients. Factors such as patient region, age, disease severity, BMI, and drug use are significantly associated with fatigue. Healthcare providers should prioritize the assessment of fatigue symptoms in these patients. Following the initial assessment, targeted interventions should be implemented to alleviate and improve these symptoms.

## 1 Introduction

Inflammatory bowel disease (IBD) is a chronic, nonspecific intestinal inflammatory disease that mainly involves ulcerative colitis (UC) and Crohn's disease (CD) (1). Both UC and CD are incurable chronic diseases that can cause chronic inflammatory changes in the gastrointestinal tract (2, 3).In recent years, the global diagnosis rate of children and adolescents has been increasing, with high-risk onset ages between 15 and 35 years (4). Approximately 25% of cases are diagnosed before the age of 18 years (1), with more severe and faster developing intestinal infections observed in the pediatric population (2). IBD is a chronic intestinal disease that negatively affects both the mental health and quality of life of afflicted children (5). Although the disease may resolve after treatment, fatigue continues to affect the patient's daily life. Fatigue is the most annoying symptom in patients with inactive disease and the second most annoying symptom in patients with active disease (3).

Fatigue refers to subjectively continuous tiredness and a lack of energy and exhaustion, which reduces one's physical and mental activities and cannot be relieved through prolonged sleep (6). As reported, 86% of people with moderate to severe IBD experience fatigue, with fatigue remaining the most exhausting symptom even in remission and garnering more attention from patients relative to bowel symptoms (7). The Clinical Guidelines for the Management of IBD published in 2021 propose that fatigue cannot be ignored because it has a serious negative impact on people's quality of life (8). It affects physical, emotional, cognitive and social functioning and affects quality of life (9–11). The International Organization for Study of Inflammatory Bowel Diseases has reached a consensus stating that numerous IBD patients have reported a notable decrease in physical activity compared to their pre-diagnosis levels, attributing this to perceived fatigue (12). A study, which surveyed the levels of physical activity among 219 individuals diagnosed with inflammatory bowel disease (IBD), revealed a notably alarming proportion of inactivity among IBD patients, specifically 94 individuals (42.9%) (13). Exercise is a potential intervention for fatigue and a subsequent decrease in their physical activity levels has been found to exacerbate fatigue even further (14, 15). However, fatigue is often overlooked by healthcare professionals when assessing the severity of symptoms or outcomes of many diseases. The existing studies concerning the fatigue of patients with IBD lack population data for children in China. Hence, the aim of this study was to describe the prevalence and degree of fatigue in a cohort of pediatric IBD patients in China. In addition, we wanted to explore the possible associations between fatigue and/or markers of disease activity.

## 2 Methods

### 2.1 Study setting and sampling

The study was conducted in the Department of Gastroenterology of Children's Hospital of Nanjing Medical University.

Patients diagnosed with IBD were recruited via convenience sampling methods from the Department of Gastroenterology of Children's Hospital from 1 September 2021 until 31 November 2022. A total of 110 questionnaires were sent out onsite in this study, and 105 were effectively received, with an effective recovery rate of 95.45%. This study was performed in accordance with the principles of the Declaration of Helsinki. The ethics committee of Nanjing Children's Hospital affiliated with Nanjing Medical University approved the study protocol on 29 August 2023. The ethical number is 202302043-1.

### 2.2 Inclusion and exclusion criteria

The inclusion criteria were being aged 5–18 years (consideration the factors of understanding, expressive capabilities, as well as the extent of admissions handled by pediatric medical institutions), having been diagnosed with IBD according to the expert consensus on the diagnosis and management of pediatric inflammatory bowel disease for more than one year (16), being able to understand and speak Chinese and having no history of cognitive impairment. The exclusion criteria were other conditions that inherently cause fatigue, independent of IBD or unwillingness to participate.

### 2.3 Instruments with validity and reliability

#### 2.3.1 General information questionnaire

A self-designed questionnaire was used, which included sex, age, geographical location, education status, source of medical expenses, duration of illness (/year), disease type, severity of disease [patients were asked about their number of bowel movements, presence of abdominal pain, presence of blood with defecation, and weight loss as mainly clinical symptoms combined pediatric ulcerative colitis activity index (PUCAI) for UC patients and pediatric Crohn's disease activity index (PCDAI) for CD patients.) (17, 18), complications (including gastrointestinal bleeding, enterobrosis, colon cancer or thrombus), body mass index (BMI) and medication use.

#### 2.3.2 Multidimensional fatigue scale (MFS)

The Multidimensional Fatigue Scale (MFS) was developed by Varni et al. (19) and later cross-culturally adapted by Bu Xiuqing et al. (20). It mainly measures the feelings of fatigue of children with chronic diseases in the past month, including three versions of 5–7 years old, 8–12 years old and 13–18 years old, which are composed of three dimensions of general fatigue, sleep fatigue and cognitive fatigue, with a total of 18 items. It was reverse scored with the Likert level 5 scoring method: 0 = 100 points (never happened), 1 = 75 points (almost never happened), 2 = 50 points (sometimes happened), 3 = 25 points (often happened), and 4 = 0 points (always happens). The lower the score is, the greater the degree of fatigue. The Cronbach's *α* coefficients of the three versions of the scales used in this study were 0.835, 0.842, and 0.924.

### 2.4 Data collection

The researchers explained the purpose and completion requirements of the study to the IBD patients and their parents. The patients completed the general information questionnaire with the help of their parents and completed the MFS by themselves. For those with poor reading ability, the researchers read the questionnaire item by item to help them fill in, avoiding the use of suggestive language. The questionnaire was completed on the spot and returned to ensure its reliability. Before submitting the questionnaire, the healthcare professionals on duty will conduct a cross-verification of this information with the medical records. In the event of any discrepancies, the medical records shall be deemed as the authoritative source.

### 2.5 Data analysis

The data were entered into EpiData 3.1 to ensure accuracy. SPSS 23.0 statistical software was used to analyze the data. The measurement data with a normal distribution were described by $\bar{x}\pm s$, and the counting data were represented by the frequency, constituent ratio and rate. T tests, ANOVA and multiple linear regression were used to analyze the influencing factors of fatigue in children with IBD, and *p* < 0.05 was considered to indicate statistical significance. Sample size calculation was conducted using G*Power software (21), with the statistical significance value accepted at *p* < 0.05 (two-tailed), setting an alpha error at 0.05, a statistical power at 0.8, and an effect size at 0.3. Taking into account a potential dropout rate of 20%, the final sample size was determined to be no less than 99 participants. Consequently, this study ultimately included a total of 110 cases.

## 3 Results

All 105 (100%) distributed questionnaires were valid. Thus, the study included 105 participants, 71 (67.62%) of whom were male. Furthermore, 95 (90.48%) patients had CD. The age of the participants ranged from 6 to 18 years, with a median (Me) of 16 years (interquartile ranges 14–18). Table 1 shows the demographics of the study sample.

**Table 1:** Demographic data of cases.

Characteristic	Children with IBD *N* (%)	$\bar{\chi }\pm s$	Statistics	*P* values
Gender			*T* = −0.354	0.724
Male	71 (67.62)	62.72 ± 20.57		
Female	34 (32.38)	61.19 ± 20.76		
Location			*T* = 2.261	0.026
Rural	20 (19.05)	53.06 ± 23.55		
Urban	85 (80.95)	64.38 ± 19.30		
Education status			*F* = 3.241	0.043
Primary and below	16 (15.24)	67.71 ± 21.05		
Junior high school	46 (43.81)	65.88 ± 19.72		
High school and above	43 (40.95)	56.27 ± 20.20		
Source of medical expenses			*T* = 1.148	0.254
Self-paying	45 (42.86)	59.57 ± 20.42		
Medical insurance	60 (57.14)	64.21 ± 20.59		
Duration of illness (/year)			*F* = 0.808	0.449
1–3	59 (56.19)	64.31 ± 20.08		
3–5	42 (40.00)	59.99 ± 21.56		
>5	4 (3.81)	54.86 ± 15.94		
Disease types			*T* = 0.179	0.858
CD	95 (90.48)	62.11 ± 21.41		
UC	10 (9.52)	63.33 ± 9.33		
Severity of disease			*F* = 6.211	0.003
Mild	40 (38.10)	69.17 ± 18.27		
Moderate	49 (46.67)	60.83 ± 19.78		
Severe	16 (15.24)	49.13 ± 22.10		
Complications			*T* = −1.793	0.076
Existent	89 (84.76)	66.67 ± 15.49		
Nonexistent	16 (15.24)	59.38 ± 22.88		
Glucocorticoid			*T* = 3.001	0.003
Use	27 (25.71)	65.63 ± 20.05		
Nonuse	78 (74.29)	52.37 ± 19.04		
Immunosuppressant			*T* = 0.811	0.419
Use	52 (49.52)	60.58 ± 20.75		
Nonuse	53 (50.48)	63.84 ± 20.41		
Biological agents			*T* = 0.070	0.006
Use	76 (72.38)	66.29 ± 19.23		
Nonuse	29 (27.62)	55.04 ± 21.07		
Enteral nutrition			*T* = 0.939	0.350
Use	66 (62.86)	60.77 ± 19.81		
Nonuse	39 (37.14)	64.67 ± 21.77		
Per capita annual household income (/thousand) (RMB)			*F* = 1.650	0.197
<30	36 (34.29)	58.26 ± 21.85		
30–50	27 (25.71)	67.70 ± 19.39		
>50	42 (40.00)	62.10 ± 19.77		
BMI			*F* = 0.888	0.415
<18.5	49 (46.67)	59.41 ± 21.54		
≥18.5, <24	48 (45.71)	64.41 ± 20.17		
≥24	8(7.62)	66.32 ± 15.81		

The total score of fatigue in children and adolescents with inflammatory bowel disease was 62.22 ± 20.55, with the lowest score of 11 and the highest score of 100. Among the three dimensions of the questionnaire, the highest average score was 65.16 ± 28.75, which was achieved for the “cognitive fatigue” dimension, whereas the “sleep/rest fatigue” dimension received the lowest average score (60.63 ± 20.07). The level of “general fatigue” was 60.87 ± 24.68 (Table 2).

**Table 2:** Total and individual scores for fatigue dimensions.

Categories	Items	Full marks	Lowest mark	Highest mark	$\bar{\chi }\pm s$
General fatigue	6	100	0	100	60.87 ± 24.68
Sleep/rest fatigue	6	100	0	100	60.63 ± 20.07
Cognitive fatigue	6	100	0	100	65.16 ± 28.75
Total score	18	100	11	100	62.22 ± 20.55

The results of the univariate analysis of the degree of fatigue in patients with IBD revealed significant differences according to region, age, disease severity, and use of corticosteroids and biological agents (*p* < 0.05). No significant differences were found in gender, source of medical expenses, disease course, disease type, immunosuppressants, enteral nutrition use, or per capita annual household income (*p* > 0.05) (Table 1).

Taking total fatigue as the dependent variable, area, education level, disease severity and glucocorticoid application, and biologic application were significantly different in the univariate analysis, and BMI, which has clinical significance, was incorporated into the multivariate analysis. Reference variables were set for education level, disease severity and body weight, with education status as primary school, disease severity as mild and body weight as reference variables (Table 3). The results revealed that urban residence, nonuse of glucocorticoids and use of biological agents were protective factors against fatigue (*p* < 0.05), whereas malnutrition, severe disease severity and high school education or above were risk factors for fatigue (*p* < 0.05) (Table 4).

**Table 3:** Independent variable assignment methods.

Independent variable	Methods
Gender	male = 0, female = 1
Location	rural = 0, urban = 1
Source of medical expenses	self-paying = 0, medical insurance = 1
Duration of illness (/year)	1–3 = 0, 3–5 = 1, >5 = 2
Disease types	CD = 0, UC = 1
Severity of disease	mild = 0, moderate = 1, severe = 2
Complications	existence = 1, inexistence = 2
Glucocorticoid	nonuse = 0, use = 1
Immunosuppressant	nonuse = 0, use = 1
Biological agents	nonuse = 0, use = 1
Enteral nutrition	nonuse = 0, use = 1
Per capita annual household income(/thousand) (RMB)	<30 = 0, 30∼50 = 1, >50 = 2
BMI	BMI < 18.5 = 0, 18.5 ≤ BMI < 24 = 1, BMI > 24 = 2

**Table 4:** Multifactor analysis of factors affecting fatigue.

	Regression coefficient	Standardized regression coefficient	*t*	*p*
Location	−6.804	−.181	−2.127	.036
Glucocorticoid	−7.078	−.210	−2.402	.018
BMI > 24	.415	.007	.085	.932
BMI < 18.5	5.748	.195	2.047	.043
Moderate	6.857	.232	2.438	.017
Severe	11.108	.271	2.887	.005
Junior high school	2.013	.068	.531	.597
High school and above	9.511	.318	2.388	.019
Biological agents	6.033	.197	2.232	.028

*R*^2^ = 33.1, adjustment *R*^2^ = 26.7.

## 4 Discussion

### 4.1 Fatigue in children and adolescents with IBD

The total fatigue score of children with IBD was 62.22 ± 20.55, a value that differed from the results of Lucia (22). This discrepancy might be related to racial differences. Furthermore, Grossman revealed that children with CD of different races reported varying levels of anxiety and fatigue (23).

In this study, the comparison of the scores of various dimensions revealed that sleep/rest fatigue and general fatigue were at higher levels, but cognitive fatigue was at a lower level. The outcomes herein were similar to those of Marcus (11), possibly because patients were more sensitive to physical rather than psychological symptoms. In adult patients with IBD, fatigue is closely related to anxiety, depression, and other emotional symptoms (24). However, the number of relevant studies in children is insufficient, so these cases are easily ignored. These findings suggest that we should pay attention not only to physical fatigue but also to psychological fatigue when managing fatigue in IBD patients.

### 4.2 Analysis of influencing factors of fatigue in children and adolescents with IBD

#### 4.2.1 Fatigue and region

This study suggests that the degree of fatigue among children living in cities is lower than that among those living in rural areas. The reason is that the income of the urban population is higher than that of the rural population, and per capita income affects investment in health (25). The health literacy level of urban residents is generally higher than that of rural residents. Moreover, with respect to equal access to basic medical and health services in urban and rural areas, problems such as insufficient total supply and an unbalanced supply structure exist, so rural residents have relatively limited access to public health resources compared with urban residents (26). Therefore, the disease diagnosis and treatment of children living in cities are more timely. Furthermore, parents of children in cities receive more support from communities and schools than do their rural counterparts. Consequently, parents who are urban residents can better master the methods of drug application and management for their children and guide them in symptom management. These parents also pay more attention to and insist on regular follow-up and long-term control, so the fatigue management of their children is more scientific.

In addition to improving access to medical treatment in rural areas, targeted health education should be conducted for children in different areas, with a special emphasis on disease management and fatigue relief in rural children. Furthermore, greater attention should be given to the regular follow-up of children in rural areas to promote scientific symptom management and relieve their fatigue.

#### 4.2.2 Fatigue and age

Age is positively correlated with the degree of fatigue in children and adolescents, which is the same as the findings of Lucia's study of fatigue in children with IBD (22). From the perspective of social psychology, children gradually enter puberty from school age, which is a critical period of growth and development. They face great changes in body and mind and are more sensitive to changes in all aspects of body and mind (27). As the child grows, the understanding of the disease and the perception of symptoms increase. Furthermore, the hospital attendance and disease burden of IBD negatively impact school attendance and then cause school difficulties, consequently producing greater psychological stress to children (28). Negative emotions affect their disease management and aggravate their fatigue.

The needs of children with IBD must be addressed by ensuring effective partnerships between education and health and targeting those with risk factors for poor attendance with preventative measures. The emotional and mental states of adolescent children must be emphasized, and the use of strategies to minimize the healthcare burden and provide more integrated care can directly impact service provision.

#### 4.2.3 Fatigue and drug use

Children treated with biologics had less fatigue than did those not treated with biologics. This finding is similar to the results of the study by Borren (29) which examined the longitudinal trajectory of fatigue in patients who started biotherapy for more than one year and confirmed that fatigue improved with the start of biotherapy and the relief of clinical symptoms. At present, the only biological agent approved for clinical use in China is infliximab, a medication that mainly targets soluble and transmembrane tumor necrosis factor (TNF)-*α*, which is a powerful proinflammatory cytokine that plays a role in the dysregulation of the mucosal immune response in IBD (30). Thus, infliximab may alleviate fatigue through cytokine action and may also alleviate fatigue by improving clinical symptoms in children.

Children who used glucocorticoids had greater degrees of fatigue than those who did not use them did, and this result was also reached by van Langenberg et al. (31). This finding might be related to the serious side effects of corticosteroids. Corticosteroid treatment can lead to adrenal insufficiency in IBD patients, affect the healing of peptic ulcers, and increase the risk of respiratory tract infection and sepsis (32). Therefore, in the future, in the management of fatigue symptoms in children and adolescents, attention should be given to drug use in children and timely treatment of drug side effects.

#### 4.2.4 Fatigue and BMI

BMI is a reliable indicator of protein energy malnutrition and is a simple and feasible method for screening for malnutrition. According to their BMI grades, 46.67% of the children with IBD were malnourished. The present study revealed that the fatigue of malnourished children is more serious than that of other children, an outcome that is the same as that reported by Whelan et al. (33). IBD is associated with anorexia. Typical symptoms include abdominal pain, diarrhea and vomiting. These symptoms further lead to discomfort and loss of appetite. IBD in children often leads to changes in dietary behavior due to hospitalization and dietary restrictions to control gastrointestinal symptoms. By hindering dietary intake, IBD symptoms further aggravate the symptoms of fatigue. Furthermore, many deficiencies in micronutrients, such as iron (34) and vitamin B12 (35), occur in children with IBD. These trace elements are closely related to fatigue symptoms. Therefore, clinical guidelines emphasize the nutritional status of children with IBD and recommend enteral nutrition therapy as a first-line dietary source of CD-induced remission in mild to moderate IBD in children by highlighting regular detection and timely correction of micronutrient levels (33). However, in this study, there was no significant correlation between the use of enteral nutrition and symptoms of fatigue in children. The reason may be related to the time and preparation of enteral nutrition or the size of the sample. A large sample study is needed to determine the relationship between the use or duration of enteral nutrition and symptoms of fatigue in children.

#### 4.2.5 Fatigue and disease severity

Our investigation revealed that children with moderate to severe IBD severity are more fatigued than are children with mild severity, an outcome that is similar to that of Pellino (36) but differs from that of Chavarría (37). The sample size of the study and research tool have an impact on the results of the study. Severe diseases are often accompanied by severe clinical symptoms, such as abdominal pain and diarrhea, and the severity of the disease is closely related to children's anxiety, depression, and loneliness (38), indicating that mental factors can directly affect the symptoms of fatigue. Therefore, the severity of illness affects children's fatigue symptoms both physically and psychologically.

### 4.3 Strengths and limitations of the work

This study directly evaluated fatigue and related variables from the perspective of children and identified multiple factors related to fatigue in IBD patients, providing a reference for further research and eventual clinical application. This work suggested that in addition to actively treating diseases and managing concurrent mental disorders, attention should also be given to children's nutritional status and understanding of diseases, and personalized management methods should be provided.

There are several limitations in this study. First, a cross-sectional survey was adopted in the study. Owing to the drawbacks of the survey method, the causal relationship cannot be determined. In addition, owing to the small sample size, the study results may be affected by confounding factors. This study only investigated 110 cases of pediatric IBD, exceeding the minimum sample size of 82 calculated by G*Power software. However, it is still a small sample study. A small sample size may give rise to significant random errors and elevated false-negative rates, which could result in some fatigue-associated factors being overlooked and excluded from the analysis. The distribution of CDs vs. UCs in the sample is another limitation of this study. This study employed a stringent sampling methodology, encompassing all children with IBD who fulfilled the inclusion criteria from September 1, 2021, to November 31, 2022. This approach ensured the absence of any selection bias. Future studies with larger sample sizes and multiple centers are needed to confirm the relevant conclusions.

## 5 Conclusion

Fatigue is common in IBD patients, and several factors contributed to fatigue in our study. To date, the mechanism of fatigue is not clear, and there is no scientific and effective systematic management mode. Therefore, after a comprehensive understanding of fatigue symptoms and their influencing factors in patients with different types of IBD, systematic, comprehensive and targeted interventions should be developed to help patients maximize the improvement of fatigue symptoms, help patients return to society and improve their quality of life.

## Data Availability

The original contributions presented in the study are included in the article/Supplementary Material, further inquiries can be directed to the corresponding author.
