# Supplementary material for: Fatigue in children and adolescents with inflammatory bowel disease: a cross-sectional study
Source: Front Pediatr. 2024 Dec 19;12:1519779. doi: 10.3389/fped.2024.1519779 (PMC11693721; doi:10.3389/fped.2024.1519779)
Supplement: Supplementary file 1 [file Table1.pdf]

S1 Table. Multidimension Fatigue Scale (MFS) Chinese version（5~7 years old）

全身疲乏	从不😀	有时有😐	经常😟
1. 你感觉累吗?	0	2	4
2. 你感觉身体虚弱(不强壮)吗?	0	2	4
3. 你感觉很累以至于不能做你喜欢做的事情吗?	0	2	4
4. 你感觉很累以至于不能和你的朋友在一起吗?	0	2	4
5. 你完成一些事情有困难吗?	0	2	4
6. 你开始做一些事情有困难吗?	0	2	4
睡眠/休息方面疲乏	从不😀	有时有😐	经常😟
1. 你睡觉很多吗?	0	2	4
2. 你很难一觉到天亮吗?	0	2	4
3. 你早上醒来的时候觉得身体很累吗?	0	2	4
4. 你休息很多吗?	0	2	4
5. 你小睡很多次吗?	0	2	4
6. 你躺在床上的时间很长吗?	0	2	4
认知疲乏	从不😀	有时有😐	经常😟
1. 你很难保持注意力在一些事情上吗?	0	2	4
2. 你很难记住别人对你讲了什么吗?	0	2	4
3. 你很难记住你刚刚听到了什么吗?	0	2	4
4. 你很难快速地思考吗?	0	2	4
5. 你很难记起刚才在想什么吗?	0	2	4
6. 你很难在同一时间记住一件以上的事情吗?	0	2	4

Chinese version（8~18 years old）

全身疲乏	从来没有	几乎没有	有时有	经常有	总是有
1. 你感觉累吗?	0	1	2	3	4
2. 你感觉身体虚弱(不强壮)吗?	0	1	2	3	4
3. 你感觉很累以至于不能做你喜欢做的事情吗?	0	1	2	3	4
4. 你感觉很累以至于不能和你的朋友在一起吗?	0	1	2	3	4
5. 你完成一些事情有困难吗?	0	1	2	3	4
6. 你开始做一些事情有困难吗?	0	1	2	3	4
睡眠/休息方面疲乏	从来没有	几乎没有	有时有	经常有	总是有
1. 你睡觉很多吗?	0	1	2	3	4
2. 你很难一觉到天亮吗?	0	1	2	3	4
3. 你早上醒来的时候觉得身体很累吗?	0	1	2	3	4
4. 你休息很多吗?	0	1	2	3	4
5. 你小睡很多次吗?	0	1	2	3	4
6. 你躺在床上的时间很长吗?	0	1	2	3	4
认知疲乏	从来没有	几乎没有	有时有	经常有	总是有
1. 你很难保持注意力在一些事情上吗?	0	1	2	3	4
2. 你很难记住别人对你讲了什么吗?	0	1	2	3	4
3. 你很难记住你刚刚听到了什么吗?	0	1	2	3	4
4. 你很难快速地思考吗?	0	1	2	3	4
5. 你很难记起刚才在想什么吗?	0	1	2	3	4
6. 你很难在同一时间记住一件以上的事情吗?	0	1	2	3	4

English version(5~7 years old)

General Fatigue	Never	Sometimes	Often
1. Do you feel tired?	0	2	4
2. Do you feel weak (not strong)?	0	2	4
3. Do you feel so tired that you cant do what you like?	0	2	4
4. Are you feeling so tired that you cant be with your friends?	0	2	4
5. Do you have difficulty completing something?	0	2	4
6. Do you have difficulty starting something?	0	2	4
**Sleep Fatigue**	**Never**	**Sometimes**	**Often**
1. Do you sleep a lot?	0	2	4
2. Is it hard for you to sleep until dawn?	0	2	4
3. Do you feel very tired when you wake up in the morning?	0	2	4
4. Have you had a lot of rest?	0	2	4
5. Have you taken many aps?	0	2	4
6. Do you stay in bed for a long time?	0	2	4
**Cognitive Fatigue**	**Never**	**Sometimes**	**Often**
1. Is it hard to keep your mind on something?	0	2	4
2. Is it hard to remember what someone else said to you?	0	2	4
3. Is it hard to remember what you just heard?	0	2	4
4. Is it hard for you to think quickly?	0	2	4
5. Can you hardly remember what you were just thinking about?	0	2	4
6. Is it hard for you to remember more than one thing at the same time?	0	2	4

English version(8~18 years old)

General Fatigue	Never	Almost never	Sometimes	Often	Always
1. Do you feel tired?	0	1	2	3	4
2. Do you feel weak (not strong)?	0	1	2	3	4
3. Do you feel so tired that you can not do what you like?	0	1	2	3	4
4. Are you feeling so tired that you can not paly with your friends?	0	1	2	3	4
5. Do you have difficulty completing something?	0	1	2	3	4
6. Do you have difficulty starting something?	0	1	2	3	4
**Sleep Fatigue**	**Never**	**Almost never**	**Sometimes**	**Often**	**Always**
1. Do you sleep a lot?	0	1	2	3	4
2. Is it hard for you to sleep until dawn?	0	1	2	3	4
3. Do you feel very tired when you wake up in the morning?	0	1	2	3	4
4. Have you had a lot of rest?	0	1	2	3	4
5. Have you taken many aps?	0	1	2	3	4
6. Do you stay in bed for a long time?	0	1	2	3	4
**Cognitive Fatigue**	**Never**	**Almost never**	**Sometimes**	**Often**	**Always**
1. Is it hard to keep your mind on something?	0	1	2	3	4
2. Is it hard to remember what someone else said	0	1	2	3	4
3. Is it hard to remember what you just heard?	0	1	2	3	4
4. Is it hard for you to think quickly?	0	1	2	3	4

5. Can you hardly remember what you were just thinking about?	0	1	2	3	4
6. Is it hard for you to remember more than one thing at the same time?	0	1	2	3	4
